# Patient safety work in Sweden: quantitative and qualitative analysis of annual patient safety reports

**DOI:** 10.1186/s12913-016-1350-5

**Published:** 2016-03-21

**Authors:** Mikaela Ridelberg, Kerstin Roback, Per Nilsen, Siw Carlfjord

**Affiliations:** Department of Medical and Health Sciences, Division of Health Care Analysis, Linköping University, SE-581 83 Linköping, Sweden; Department of Medical and Health Sciences, Division of Community Medicine, Linköping University, SE-581 83 Linköping, Sweden

**Keywords:** Healthcare, Patient safety, Patient safety reports

## Abstract

**Background:**

There is widespread recognition of the problem of unsafe care and extensive efforts have been made over the last 15 years to improve patient safety. In Sweden, a new patient safety law obliges the 21 county councils to assemble a yearly patient safety report (PSR). The aim of this study was to describe the patient safety work carried out in Sweden by analysing the PSRs with regard to the structure, process and result elements reported, and to investigate the perceived usefulness of the PSRs as a tool to achieve improved patient safety.

**Methods:**

The study was based on two sources of data: patient safety reports obtained from county councils in Sweden published in 2014 and a survey of health care practitioners with strategic positions in patient safety work, acting as key informants for their county councils. Answers to open-ended questions were analysed using conventional content analysis.

**Results:**

A total of 14 structure elements, 31 process elements and 23 outcome elements were identified. The most frequently reported structure elements were groups devoted to working with antibiotics issues and electronic incident reporting systems. The PSRs were perceived to provide a structure for patient safety work, enhance the focus on patient safety and contribute to learning about patient safety.

**Conclusion:**

Patient safety work carried out in Sweden, as described in annual PSRs, features a wide range of structure, process and result elements. According to health care practitioners with strategic positions in the county councils’ patient safety work, the PSRs are perceived as useful at various system levels.

**Electronic supplementary material:**

The online version of this article (doi:10.1186/s12913-016-1350-5) contains supplementary material, which is available to authorized users.

## Background

It was over 2000 years ago that Hippocrates said “first, do no harm”, yet until recently medical errors were considered an inevitable consequence of health care. There is now widespread recognition of the problem of unsafe care and tremendous efforts have been made over the last 15 years to improve patient safety. Like many other countries, Sweden has seen increased patient safety activities at national, regional and local levels in recent years. A new law on patient safety [1] was introduced in 2011 along with a government-supported financial incentive plan whereby over two billion SEK has been allocated during 2011–2014 to county councils (21 regional administrative authorities in Sweden which have autonomy regarding the provision of health care services to their citizens) that carry out specific patient safety-enhancing activities and achieve certain results with regard to patient safety. For details regarding the financial incentive plan, see Additional file 1.

The new patient safety law in Sweden obliges all county councils to assemble a yearly patient safety report (PSR), a written document describing the patient safety work that has been conducted in primary and hospital care, and what results have been achieved in the previous calendar year. The aim is to provide a comprehensive picture of the patient safety work, allowing for tracking of progress over time and identifying areas for improvement. PSRs have been published since 2011. A template for the PSR was introduced in 2012 [see Additional file 2], recommending that the patient safety work should be documented in accordance with Donabedian’s [2] triad of structure (organization, infrastructure and general conditions for patient safety work), process (patient safety-related activities carried out) and results (patient safety-related measures). We have not been able to identify any studies in the patient safety literature describing this sort of documentation on patient safety work. The Hospital Safety Score in the US [3] is another effort to assemble information about patient safety work and results although it is a survey rather than a narrative document as the Swedish PSR. There might also be other examples but to our knowledge the PSRs seems to be unique.

The county councils’ PSRs could potentially facilitate efforts for improved patient safety at the regional level and provide relevant information for coordinated national efforts to achieve safer care. However, the extent to which such potential advantages of the PSRs are realized has not been assessed. It is important to evaluate the PSRs in terms of how extensive the reporting is and the perceived usefulness of these reports. Therefore, the aim of this study was to describe the patient safety work carried out in Sweden by analysing the PSRs with regard to the structure, process and result elements reported in 2014. The aim was also to investigate the usefulness of the PSRs as a tool to achieve improved patient safety, as perceived by health care practitioners with strategic positions in patient safety work in the county councils in Sweden.

## Methods

### Study setting

The study was conducted in Sweden. The Swedish health care system is organized at national, regional and local levels. At the regional level, the responsibility for financing and providing health care is decentralized to 21 county councils. The county councils have full budgetary responsibility for providing health care to all citizens in their region. The Swedish health care system is primarily funded by taxes. This is supplemented by government grants and, to a small extent, by patient fees.

There are many actors in Swedish health care that influence the county councils’ work in different ways. An important national actor from a patient safety perspective is the Swedish Association of Local Authorities and Regions (SALAR), a members’ organisation for all the county councils. SALAR represents the county councils in dialogue with the Swedish Government. The National Board of Health and Welfare (NBHW), a government agency under the Ministry of Health and Social Affairs, produces and provides recommendations and regulations for health and social care personnel and managers and monitors the quality of care delivered in Sweden. A task of the Health and Social Care Inspectorate is to supervise health and medical care. Incidents that have led or could have led to serious health damage must be reported to the Health and Social Care Inspectorate according to the law Lex Maria. On a regional and local level there are Patients’ Advisory committees, assigned to give support and guidance to patients, relatives and staff regarding issues on publicly financed health care.

### Data sources

The study was based on two sources of data: PSRs obtained from county councils in Sweden published in 2014 and a survey of health care practitioners with strategic positions in patient safety work, acting as key informants for their county councils.

### Patient safety reports – description and analysis

To obtain data for the present study, we collected the PSRs from the county councils’ websites within 2 weeks of their publication (1 March 2014). The county councils that did not have a PSR available on their website were contacted and asked to provide the PSR.

PSRs are publicly available and intended for county council managers and health care practitioners in the county councils as well as external stakeholders such as patient organizations, authorities, patients and the general public. The first PSRs were assembled in 2011 and reports have been published annually since then. Initially, minimal guidance was provided on how the reports should be structured, resulting in inconsistent reporting and some wordy documents. A template [see Additional file 2] was introduced in 2012 to provide guidelines on what should be reported, although the county councils are not obliged to use the recommended structure. The reports have been continuously refined in a process whereby researchers have scrutinized the documentation and provided feedback and suggestions to the Swedish Association of Local Authorities and Regions (SALAR) for improvement.

The authors of the present study have analysed all PSRs published since 2011 [4]. A coding scheme was developed by the authors of the present study when the first PSRs were published in 2011. An initial inductive analysis of all published reports was performed using conventional content analysis [5]. Important information was highlighted and coded according to content, before being classified into the three categories of Donabedian’s [2] triad: structure (reported organization, infrastructure and general conditions for patient safety work), process (reported patient safety-related activities carried out) and results (reported patient safety-related measures). Donabedian’s triad is commonly used to evaluate health care quality.

The coding scheme has been iteratively modified by the authors since the first draft in 2011. Several meetings were held among the authors between 2011 and 2013 to discuss and refine the coding scheme and content of the categories. Any disagreements were resolved at these meetings. Discussions were also held with representatives of SALAR and the National Board of Health and Welfare to discuss and verify the accuracy of the categorization of the elements.

A validation of the coding scheme was performed whereby two randomly selected PSRs were read and coded independently by all the authors of the present article. A review and comparison between the authors’ coding showed few discrepancies, suggesting a high level of inter-coder reliability of the coding scheme used [6].

Applying the established coding scheme, the PSRs were read and analysed with conventional content analysis [5] by M.R., and the content was classified into the structure, process and outcome categories. Process and structure elements were assigned one of four labels: “implemented”; “partly implemented” (if all healthcare functional units in a county council had partially implemented a structure or process element or the structure/process element had been fully implemented in some units but not in all); “unclear reporting” (mentioned, but impossible to judge if implemented or not); or “not reported”. The presence of outcome elements was coded as either “yes” or “no”. Data were entered into Microsoft Excel 2010 for data management.

### Survey questionnaire – description and analysis

A survey was conducted to evaluate the perceived usefulness of the PSRs. Two open questions aimed to capture the perceived usefulness of the PSRs: “How can the PSR help to improve patient safety? Please give examples.” and “In what ways have patient safety reports been useful in your county council?”

The study population for the survey questionnaire consisted of 222 health care practitioners identified as having a strategic position in patient safety work in the county councils. They were recruited in collaboration with designated members in a SALAR patient safety network, representing all 21 county councils. These representatives were asked to identify respondents whom they considered had good knowledge and overview of the county council’s patient safety work and the ability to influence decisions concerning these efforts. The number of respondents from each county council ranged from 3 to 15; the number was proportional to the size of the population and the health care budget of the county councils.

The questionnaire was sent to the respondents by mail in June 2014 together with a stamped return envelope. All respondents received one reminder by e-mail approximately 3 weeks after the first mailing. Data from the questionnaires were entered in a Microsoft Office Access database independently by two persons.

Responses to the open questions were analysed using conventional content analysis [5] which allows the categories to emerge inductively from the data. Three of the authors (M.R., K.R. and S.C.) analysed the written answers independently using open coding to categorize the responses. During a meeting, they then categorized the findings into a number of categories. These categories were then scrutinized by the fourth author (P.N.), who suggested a few changes. Another meeting was then held among the four authors where consensus was reached. Responses to the open questions were generally brief, which meant that the answers were to the point and did not contain many expositions or elaborations. The “de-contextualized” responses made the analysis fairly straightforward.

### Ethical approval

Ethical approval was not sought for this study because it did not involve sensitive personal information, as specified in Swedish law regulating ethical approval for research concerning humans [7]. The survey participants received a cover letter with information regarding the study aim, that participation was voluntary and that data would be treated confidentially. Thus, returning the questionnaire was interpreted as informed consent.

## Results

### Results from the patient safety reports

Twenty of the 21 county councils in Sweden provided a complete PSR for 2014. One county council published a number of individual reports pertaining to different health care organizations operating within the county council. These were not included in the analysis. Tables 1, 2 and 3 show the distribution of the elements in each category as reported by the 20 county councils. The label “not reported” was omitted from the tables.

**Table 1:** Structure elements of the county councils’ patient safety work

The county council reports that it has …	Number of county councils		
	Implemented	Partly implemented	Unclear reporting
Group devoted to work with antibiotics issues according to the STRAMA concept^a^at the county council level (included in national financial incentive plan)	20	0	0
Electronic incident reporting system	19	0	1
Structure for interaction with patients and families regarding patient safety-related issues	17	2	1
Group devoted to working with hygiene issues	16	0	3
Training/education in patient safety-related issues	16	2	1
Structure for collaboration with Patients’ Advisory Committee^b^	14	1	4
Specified measurable regional patient safety goals	13	0	6
Structure for coordination and collaboration in health care transitions involving external health care providers	13	3	3
Patient safety unit that plans, carries out and evaluates patient safety work	10	2	0
System for communicating patient safety-related information to staff and patients	7	9	3
Advisory pharmaceutical committee	5	0	0
Structure for collaboration and coordination between clinics	5	5	0
System for detection and proper treatment of seriously ill patients^d^	4	3	2
National electronic system to prevent and reduce health care-associated infections and incorrect antibiotic prescriptions (the Infection Tool^c^) (included in national financial incentive plan)	3	14	2

^a^STRAMA is a multi-professional national network with the aim of achieving rational use of antibiotics in Swedish health care

^b^Patients, relatives and staff can turn to the Patients’ Advisory committee free of charge regarding issues on publicly financed health care. They give support and guidance

^c^Infection Tool is a national electronic system to prevent and reduce health care-associated infections and incorrect antibiotic prescriptions. The tool can be used to generate results for comparisons at the local, regional and national levels

^d^Examples include MEWS (Modified Early Warning Score) and RETTS (Rapid Emergency Triage and Treatment System)

**Table 2:** Process elements of the county councils’ patient safety work

The county council reports the use, work with or participation in …	Number of county councils		
	Implemented	Partly implemented	Unclear reporting
Surveys of patient safety culture (included in the national financial incentive plan)	18	0	1
Measurements of point prevalence of health care-associated infections	18	0	1
Measurements of compliance with basic hygiene and dressing rules (included in the national financial incentive plan)	18	0	1
Measurements of prevalence of pressure ulcers (included in the national financial incentive plan)	18	0	1
Action plans based on the results from the patient safety culture survey (included in national financial incentive plan)	18	0	1
Risk analyses	17	1	1
Retrospective medical record reviews such as the Global Trigger Tool (included in the national financial incentive plan)	15	2	2
Reduction and/or optimization of antibiotic prescriptions (included in the national financial incentive plan)	15	0	4
Prevention of pressure ulcers (included in the national financial incentive plan)	14	1	1
Senior Alert national quality registry^b^	14	1	1
Leadership Walk Rounds and/or other forms of patient safety dialogues that engage leaders in patient safety issues	13	3	3
Measurements of patient overcrowding (included in the national financial incentive plan)	11	0	1
Action plans based on the results of pressure ulcer measurements (included in the national financial incentive plan)	11	0	3
Root cause analyses	10	1	8
Prevention of falls	10	1	4
Prevention of malnutrition	8	2	3
Medication reviews and/or medication reports^a^	8	7	2
National measurements of patients’ perceptions of health care quality	8	5	4
Palliative Care national quality registry^c^	7	0	2
National Patient Overview^d^ (included in national financial incentive plan)	7	3	1
National electronic system for analysis and sharing of root cause analyses	6	1	4
Limiting the spread of antibiotic-resistant bacteria and other infections	6	0	2
Structured communication tools such as SBAR (Situation, Background, Assessment, Recommendation)	6	2	6
Prevention of medication-related problems, including safe and effective prescribing, handling and preparation of medications	5	2	2
Prevention of health care-associated urinary tract infections and urine catheter-related infections	5	0	1
Hygiene rounds and/or other active hygiene follow-ups	5	1	0
WHO Safe Surgery checklist	5	1	0
Improving patient safety in medical technology	5	1	1
Prevention of central intravenous line infections	4	1	1
Prevention of medication errors in health care transitions	3	1	2
Prevention of postoperative wound infections	2	1	2

^a^Medication reviews involve structured examination and safety evaluation of a patient’s medical treatment. Medical reports are a statement/explanation of changes made in medical treatment during a care episode

^b^Senior Alert is a national quality registry that documents risks regarding nutrition, falls and pressure ulcers and specifies what measures have been or will be taken to minimize these risks

^c^Palliative Care registry is a national quality registry that contains individual data concerning patient problems, medical interventions, and outcomes of treatments; within all health care settings

^d^National Patient Overview (Swedish abbreviation NPÖ) enables health care providers to access patient information from other health care providers (county councils, municipalities and private health care providers)

**Table 3:** Outcome elements of the county councils’ patient safety work

The county council reports results concerning…	Number of county councils
Health care-associated infection (HAI)	19
Compliance with basic hygiene and dressing rules (included in national financial incentive plan)	19
Pressure ulcers (included in national financial incentive plan)	18
Incidents in reporting systems	18
Lex Maria^a^	18
Complaints to the Patients’ Advisory Committee^b^	17
Antibiotic prescriptions (included in the national financial incentive plan)	16
Risk analyses	16
Complaints to the Health and Social Care Inspectorate^c^	16
Retrospective medical record reviews	14
Patient overcrowding (included in national financial incentive plan)	13
Root cause analyses	11
Complaints to the national Patient Injury Insurance agency	11
Fall injuries	8
Patient risk assessments reported in Senior Alert	8
Patient safety culture (included in national financial incentive plan)	7
Malnutrition	6
Medication reviews^d^ (included in national financial incentive plan)	6
Medication reports^d^	6
National Patient Questionnaire	5
Avoidable inpatient/hospital care	2
Availability of health care	1
Suicide assessments	1

^a^Lex Maria is a regulation for health care givers/organizations whereby incidents that have led or could have led to serious health damage must be reported to the Health and Social Care Inspectorate

^b^Patients, relatives and staff can turn to the Patients’ Advisory committee free of charge regarding issues on publicly financed health care. They give support and guidance

^c^The Health and Social Care Inspectorate now supervises health and medical care, social services and services under the Act concerning Support and Service for Persons with Certain Functional Impairments. The Inspectorate is also responsible for the consideration of permits in these areas

^d^Medication reviews involve structured examination and safety evaluation of a patient’s medical treatment. Medical reports are a statement/explanation of changes made in medical treatment during a care episode

With regard to the structure of the patient safety work, 14 different elements were identified in the PSRs (Table 1). The majority of the county councils, 14 of 20, reported between 6 and 10 structure elements in their PSRs. The most frequently reported elements were groups devoted to work with antibiotics issues according to the concept developed by STRAMA (multi-professional national network with the aim of achieving rational use of antibiotics in Swedish health care) at the county council level (reported by all county councils) and electronic incident reporting systems (19 county councils). Fourteen of the 20 county councils reported partial implementation of a national electronic system to prevent and reduce health care-associated infections and incorrect antibiotic prescriptions.

Thirty-one different process elements could be identified in the PSRs (Table 2). Half of the county councils reported between 11 and 15 process elements in their PSRs. Six county councils reported between 16 and 20 process elements and two reported 21 or more process elements. Eighteen county councils reported measurements concerning point prevalence of health care-associated infections (HAI), compliance with basic hygiene and dressing rules, and prevalence of pressure ulcers. Action plans based on the results from patient safety culture surveys were also reported by 18 county councils.

The PSRs included 23 different outcome elements (Table 3). Thirteen of the 20 county councils reported between 11 and 15 outcome elements and three reported between 16 and 20. Nineteen county councils reported results concerning HAI and compliance with basic hygiene rules and dress codes, and 18 reported results with regard to pressure ulcers, incidents in reporting systems and Lex Maria, which is a regulation for health care organizations in Sweden that requires severe incidents to be reported to the Health and Social Care Inspectorate. Two main types of outcome elements were reported in the PSRs: (1) the results of measurements linked to process elements that are included in the national financial incentive plan [see Additional file 1], such as HAI and patient safety culture measurements; (2) information intended for internal use within a county council (e.g., incident reporting) or information of external relevance for other authorities (e.g., the legally obligated reports to Lex Maria).

### Usefulness of the patient safety reports

Based on the open-answer questions of the survey an analysis of the perceived usefulness of the patient safety reports was performed. Of the 222 patient safety practitioners who received the questionnaire, 156 individuals answered the questionnaire, yielding a response rate of 70 %. Nearly three-quarters of the respondents (73 %) had been designated to work with patient safety issues in their county council for 3 years or more and approximately two-thirds (66 %) believed that they had excellent or very good knowledge of the county council’s patient safety work. Of the 156 respondents, 105 (67 %) provided answers to one or both of the open questions. Three types of perceived usefulness (i.e., categories) of the PSR emerged from the analysis of the answers: Provides a structure for patient safety work; Enhances the focus on patient safety; and Contributes to learning about patient safety.

#### PSR provides a structure for patient safety work

Respondents believed that the PSR gives an overview of the patient safety work in the county council because it summarizes this work and provides a foundation for analysis and assessment, identification of weaknesses and areas for improvement, as well as for developing action plans and initiatives. The PSR is perceived to provide valuable input for prioritizing and making decisions concerning patient safety by the top-level management and political leadership in the county councils.

#### PSR enhances the focus on patient safety

Respondents expressed the opinion that the PSR sends a strong signal to the management at different levels that patient safety work is an important priority in the county council. Documentation of patient safety work and the results in the PSR create increased awareness, inspiration and engagement, which, according to the respondents, could have a positive impact on the patient safety culture in the county council.

#### PSR contributes to learning about patient safety

Respondents described the PSR as a tool for benchmarking between county councils, as they believed the document contributes to a beneficial competition and stimulates improvement efforts. Publishing and making the PSRs available to other county councils provides a means to disseminate best practice examples and encourage reflection and dialogue on patient safety issues within units, clinics, hospitals and county councils. Respondents also mentioned the potential for citizen participation and involvement, which could lead to higher expectations for efforts to achieve improved patient safety.

## Discussion

This study sought to describe the patient safety work carried out in Sweden by analysing the PSRs and investigating the usefulness of these reports as a tool to achieve improved patient safety. The main findings were that the county councils’ PSRs feature a wide range of structure, process and outcome elements with regard to patient safety work carried out in Sweden. The PSRs demonstrate that patient safety work in many county councils is fairly comprehensive. Health care professionals with strategic positions in the county councils believed that the PSRs could improve patient safety by improving the structure of this work, enhancing the focus on patient safety and contributing to learning regarding patient safety.

Work concerning HAIs and the use of antibiotics was reported in the PSRs in numerous structure, process and outcome elements. Considering the risks associated with the use of antibiotics and antimicrobial resistance [8], the emphasis of these issues in the PSRs is not surprising. Reduction in use of antibiotics is a major area of patient safety in many countries [9–11]. Sweden has established STRAMA teams, which work towards rational use of antibiotics. In France, central infection control teams similar to the STRAMA teams have been shown to be successful in gaining institutional control over specific HAIs in a large regional multi-hospital institution [12].

Efforts to decrease HAI and the use of antibiotics in Sweden are supported by a national electronic system, called the Infection Tool. This tool requires detailed documentation when antibiotics are prescribed and provides local data on prescriptions and HAI status to enhance learning among practitioners. Implementation of the tool is expected to provide more effective support for reducing antibiotic prescriptions than the current point prevalence measures of HAI that have been carried out since 2008. Many obstacles concerning HAI data collection, analysis, dissemination and local interpretation of the results have been identified [13]. According to the PSRs the Infection Tool is not yet fully implemented over the country, but work is ongoing, potentially implying reduced antibiotic prescriptions in the near future.

The proportion of occupied beds or rate of overcrowding was frequently reported as an outcome in the PSRs, but measuring overcrowding is less frequently used as a process element. The problem with overcrowding is a well-known risk factor for HAI, although more research is needed to determine how different levels of bed occupancy correspond with the frequency of HAIs [14].

Electronic incident reporting systems have been implemented in Sweden, according to the PSRs. Reporting of incidents is mandatory for all health care professionals and has long been considered a cornerstone of patient safety work in Sweden and elsewhere. However, the effectiveness of incident reporting has recently come under a great deal of criticism in Sweden. There have even been calls for stopping incident reporting because the effect on patient safety is uncertain [15]. Numerous international researchers [16–20] have argued that reporting and analysis of incidents is insufficiently linked to appropriate action and feedback to have any impact on patient safety, advocating a shift in attention away from reporting and analysis towards how systems and instruments can be utilized for organizational learning to achieve safer care.

WHO’s Safe Surgery checklist is an instrument that has been shown to reduce rates of death and complications among patients after surgery [21]. The checklist has been widely implemented internationally and is considered to be an evidence-based surgery intervention [21–23]. Despite the research support for this intervention, only one in four of the Swedish county councils reported that they use the checklist. This implies a need for better implementation of the checklist in the future.

Structures for interaction with patients and families regarding patient safety-related issues seem to be widely implemented (reported as a structure element). However, the PSRs did not feature any corresponding process or outcome elements associated with this structure, which makes it difficult to verify how these structures for interaction are used in terms of actual activities or what the results may be. There is a strong international and national policy trend towards increased emphasis on patient involvement, but thus far no specific interventions in this area have been found to be effective [24, 25], which might provide an explanation as to why process and outcome elements are lacking. It has been increasingly recognized that patients might be an underutilized resource in efforts for improved patient safety [26].

Most of the county councils reported that they have action plans based on the results from the patient safety culture survey. These action plans were part of the current financial incentive plan, which explains the high levels of reporting. Patient safety culture is measured regularly in all county councils and the results are fed back to the local health care authorities. The action plans build on the results from the patient safety culture measurements and are intended to identify areas for improvement and describe how they will be addressed. Although there is emerging evidence to support the potential effectiveness of interventions aimed at improving safety culture [27], there is no available evidence to support that measurement of patient safety culture and development of action plans with specified target areas, as suggested in the financial incentive plan, actually improve the patient safety culture.

The usefulness of the PSR was described in favourable terms by the health care professionals with strategic positions in the county councils. The potential benefits that were identified can be attributed to different system levels, decision maker level, management level, staff level and patient level. At the decision maker level PSRs can provide input for priority setting within the county councils. The PSRs may also have a benchmarking effect by identifying good examples or best practices and contribute to generating a sense of “competition” regarding patient safety [28]. At the management level, the PSR facilitates an overview of patient safety work and provides guidance for the structure of this work. At the staff level, the PSR might contribute to emphasizing the importance of patient safety issues and could possibly influence the patient safety culture, an issue considered increasingly important in patient safety work [27]. The usefulness at the patient level could be described in terms of patient involvement and empowerment, because patients who are well informed about local patient safety activities will be in a better position to form and express their opinion on the issue [29].

### Methodological considerations

There are some limitations in this study that should be considered when interpreting the results. All the information regarding elements of structure, process and outcome is based on the published PSRs. This means that patient safety work may have been carried out, though not reported in the county councils’ PSRs. Reporting in the PSRs was not always consistent or transparent. There were individual PSRs in which an element was mentioned but it was difficult to determine whether implementation of this element was planned or had already been completed. We did not consult other sources to obtain further information if something was missing or incompletely reported.

The coding scheme might be considered a strength of the study. The scheme underwent meticulous scrutiny and was improved over the years to ascertain the validity and reliability of the analysis, including a test for inter-coder reliability. Most county councils applied the Donabedian [2] triad (structure, process, outcome) as headings for the reported elements, but the interpretation of the three categories was not always consistent. The coding scheme helped the authors to interpret the content of the reports when there were inconsistencies, e.g., reporting of the same elements under several headings and in various places in the reports.

For the questionnaire the number of informants from the county councils differed according to the size of the county council. The majority of the responders stated that they had excellent or very good knowledge of the county council’s patient safety work, and should be able to give a relevant picture. However, it is possible that further perceptions might have been expressed if a higher number of respondents had been included from the smaller county councils.

### Implications

Providing a regional-level PSR provides a means to summarize the patient safety work carried out and results achieved in a county council for both internal and external stakeholders. The quality and usefulness of PSRs could be further improved through more detailed guidelines to make the reporting more consistent and clear. PSRs may have the potential to influence patient safety work in a positive direction but further research is needed to evaluate how the documents might affect various patient safety outcomes. Wider dissemination of PSRs or similar approaches would make it possible to evaluate various aspects of the impact or effects of the use of these documents.

## Conclusions

Patient safety work carried out at the regional level in Sweden, as described in annual PSRs assembled by the county councils, features a wide range of structure, process and result elements. According to health care practitioners with strategic positions in the county councils’ patient safety work, the PSRs are useful for providing a structure for this work, enhancing the focus on patient safety and contributing to learning regarding patient safety.

## Additional files

Additional file 1: The National Financial Incentive plan for Patient Safety 2011–2014. (PDF 191 kb) Additional file 2: Headings in Patient Safety Report Template 2014. (PDF 166 kb)
