# Supplementary material for: Patient safety work in Sweden: quantitative and qualitative analysis of annual patient safety reports
Source: BMC Health Serv Res. 2016 Mar 21;16:98. doi: 10.1186/s12913-016-1350-5 (PMC4802598; doi:10.1186/s12913-016-1350-5)
Supplement: Additional file 1: — The National Financial Incentive plan for Patient Safety 2011–2014. (PDF 191 kb) [file 12913_2016_1350_MOESM1_ESM.pdf]

Additional file 1 – The National Financial Incentive Plan for Patient Safety 2011- 2014

The National Financial Incentive Plan for Patient Safety - translated by the authors (MR, PN)
Available 2015-08-20 [in Swedish]: http://skl.se/download/18.99cec5e1477430727ca1c75/1408107042312/SKL-fran-styrelsen2013-09+SB+Patients%C3%A4kerhet.pdf

2011	2012	2013	2014
Assemble an annual patient safety report (*)	Assemble an annual patient safety report (*)	Assemble an annual patient safety report (*)	Assemble an annual patient safety report (*)
Form a local STRAMA group<br><br>Work for increased adherence to local treatment recommendations, and reduce antibiotic prescribing (**)	Work for increased adherence to local treatment recommendations, and reduce antibiotic prescribing (**)	Work for increased adherence to local treatment recommendations, reduce antibiotic prescribing and provide overview of the prescriptions (**)	Work for increased adherence to local treatment recommendations, reduce antibiotic prescribing and provide overview and evaluate effects of the prescriptions (**)
Participate in the national patient survey in primary health care (*)	Reach average results regarding measures of patient-perceived quality in the national patient survey (**)	Participate in the national patient survey in primary health care (*)	Participate in the national patient survey in primary and hospital health care (*)
Make essential steps in implementation of National Patient Overview (NPO) (*)	Continue working with NPO and demonstrate wide spread use (*)	Continue working with NPO and demonstrate wider use in comparison with previous year	Continue working with NPO and demonstrate wider use in comparison with previous year
Measure the patient safety culture (**)	Make action plans and initiate improvement projects on patient safety culture (**)	Pursue and evaluate improvement projects on patient safety culture and update action plans (*)	Repeat survey of patient safety culture, evaluate improvement projects and update action plans (*)
Measure compliance with hygiene rules and dressing rules (**)	Measure compliance with hygiene rules and dressing rules and reach average compliance of 64 % (**)	Measure compliance with hygiene rules and dressing rules and reach average compliance of 70 % (**)	Measure compliance with hygiene rules and dressing rules, and reach average compliance of 72 % (**)
Measure prevalence of pressure ulcers (**)	Make action plans and initiate an improvement project regarding pressure ulcers (**)	Measure prevalence of pressure ulcers, update action plans and pursue the improvement project on pressure ulcers (**)	Measure prevalence of pressure ulcers, update action plans, pursue the improvement project and reach goals for recommended actions (**)
	Initiate implementation of national electronic system to prevent and reduce health care-associated	Make essential steps in implementation of the Infection Tool (**)	Implement the Infection Tool in specified range and validate data quality (**)

	infections and incorrect antibiotic prescriptions, the ”Infection Tool” (*)		
	Work systematic with retrospective medical records reviews at all hospitals (*)	Continue to work with and develop retrospective medical records reviews (*)	Continue to work with and develop retrospective medical records reviews (*)
	Measure patient overcrowding according to set definitions (**)	Measure patient overcrowding according to set definitions (**)	Measure patient overcrowding according to set definitions and establish an action plan (**)
		Establish routines and start an improvement project concerning information on medications at discharge from hospital (**)	Implement routines regarding medications at discharge and start monitoring compliance (**)
(*) = basic (mandatory) requirements to be eligible for reimbursement<br>(**) = performance-based requirements			
