# Supplementary material for: Patient safety work in Sweden: quantitative and qualitative analysis of annual patient safety reports
Source: BMC Health Serv Res. 2016 Mar 21;16:98. doi: 10.1186/s12913-016-1350-5 (PMC4802598; doi:10.1186/s12913-016-1350-5)
Supplement: Additional file 2: — Headings in Patient Safety Report Template 2014. (PDF 166 kb) [file 12913_2016_1350_MOESM2_ESM.pdf]

Additional file 2 – Headings in PSR Template 2014

**Headings in Patient Safety Report Template 2014** – translated by the authors (MR, PN) http://skl.se/halsasjukvard/patientsakerhet/systematisktpatientsakerhetsarbete/patientsakerhetsberattelse.988.html
• Summary<br>• Overall goals and strategies<br>• Organisational responsibility for patient safety<br>• Structure for monitoring/evaluation of the patient safety work<br>• Follow-up through self-control<br>• How has the patient safety work been carried out and what measures have been taken for increased patient safety?<br>• Collaboration to prevent adverse events<br>• Risk analysis<br>• Health professionals' reporting responsibility<br>• Handling of complaints<br>• Compilation and analysis<br>• Collaboration with patients and families<br>• Results<br>• Overall objectives and strategies for the coming years
